# Development and validation of a multivariable nomogram predictive of hepatitis B e antigen seroconversion after pregnancy in hepatitis B virus-infected mothers

**DOI:** 10.3389/fmed.2024.1428569

**Published:** 2024-10-21

**Authors:** Wenting Zhong, Jie Zheng, Che Wang, Lei Shi, Yingli He, Yingren Zhao, Tianyan Chen

**Affiliations:** ^1^Department of Infectious Disease, The First Affiliated Hospital of Xi’an Jiaotong University, Xi’an, China; ^2^Department of Radiology, The First Affiliated Hospital of Xi’an Jiaotong University, Xi’an, China; ^3^Department of Radiology Oncology, The First Affiliated Hospital of Xi’an Jiaotong University, Xi’an, China

**Keywords:** HBV, HBeAg seroconversion, postpartum therapy, nomogram, HBV-infected mother, MTCT, pregnancy hepatitis flare, prediction model

## Abstract

**Background and aims:**

Current guidelines are controversial regarding the continuation of nucleos(t)ide analogues (NAs) therapy after delivery in Hepatitis B virus (HBV)-infected pregnant women. The postpartum period may be an opportune moment for achieving hepatitis B e antigen (HBeAg) seroconversion earlier with constant NAs therapy due to the restoration of immune function after delivery. We investigated prenatal and pregnant factors associated with HBeAg seroconversion after pregnancy and developed a nomogram to predict HBeAg seroconversion rates, aiding decision-making for optimal management in women.

**Methods:**

We retrospectively included 489 HBeAg-positive mothers as the training cohort from January 2014 to December 2018 and prospectively enrolled 94 patients as the external validation cohort from January 2019 to December 2021 at the First Affiliated Hospital of Xi’an Jiaotong University. In the training cohort, independent predictors were identified using the least absolute shrinkage and selection operator (LASSO) regression algorithm. Subsequently, multivariate logistic regression was employed to establish the nomogram. Model performance was assessed using the area under the receiver operating characteristic curve (AUC), calibration plots, and decision curve analysis (DCA). Both discrimination and calibration were evaluated through bootstrapping with 1,000 resamples. The external validation cohort was subsequently used to validate the nomogram.

**Results:**

Factors such as pregnancy hepatitis flare (OR: 5.122, 95% CI: 2.725–9.928, *p* < 0.001), NAs therapy after delivery (OR: 15.051, 95% CI: 6.954–37.895, *p*: <0.001), hepatitis B surface antigen (HBsAg) (OR: 0.549, 95% CI: 0.366–0.812, *p*: 0.003) and HBV DNA level at delivery (OR: 0.785, 95% CI: 0.619–0.986, *p*: 0.041) were included in the final risk model. The AUC in the training set was 0.873 (95% CI: 0.839–0.904). The calibration curve of the nomogram closely resembled the ideal diagonal line. DCA showed a significantly better net benefit in the model. External validation also confirmed the reliability of the prediction nomogram. The AUC in the external validation set was 0.889 (95% CI: 0.801–0.953). The calibration curve for the external validation set was in close proximity to the ideal diagonal line. DCA also demonstrated a significant net benefit associated with the predictive model, consistent with the findings in the training set. Finally, the nomogram has been translated into an online risk calculator that is freely available to the public (https://wendyzhong.shinyapps.io/DynNomapp/).

**Conclusion:**

We developed a nomogram based on prenatal and pregnant factors to estimate HBeAg seroconversion after delivery in women. This tool provides clinicians with a precise and effective way to identify individuals likely to undergo HBeAg seroconversion postpartum, aiding in decision-making for optimal management.

## 1 Introduction

Hepatitis B virus (HBV) infection constitutes a global health concern, affecting approximately 296 million individuals worldwide and serving as the primary causative factor behind cirrhosis and hepatocellular carcinoma (HCC) (1).

Hepatitis B e antigen (HBeAg) seroconversion is a vital serological milestone in the context of chronic HBV infection. It is often accompanied by a reduction in hepatitis B virus (HBV) DNA levels, hepatitis B surface antigen (HBsAg), alanine aminotransferase (ALT) reversion, and disease remission (2, 3). According to a meta-analysis (4), patients who undergo HBeAg seroconversion exhibit a markedly lower overall incidence of HCC at 3.33% compared to those with persistent HBeAg positivity. Furthermore, achieving early HBeAg seroconversion further diminishes the likelihood of HCC.

However, HBeAg seroconversion rates were lower in the immune-tolerant (IT) population (5). Even with nucleos(t)ide analogues (NAs) therapy, the annual HBeAg seroconversion rate remains below 5% (6, 7). It turns out that unlike the general IT population, postpartum women with HBV may experience a higher rate of HBeAg seroconversion due to hormonal fluctuations and immune system restoration following childbirth (8–11). A study conducted by Lin et al. (10) revealed that even in the absence of NAs, approximately 12.5% of women achieve this target around one year postpartum. Furthermore, multiple studies have shown that (11, 12) continuing antiviral therapy (AVT) treatment after delivery can achieve higher HBeAg seroconversion rates. The postpartum period may offer a favorable window for achieving HBeAg seroconversion. However, there is still controversy about whether to continue treatment for this population after childbirth, especially for pregnant women in the IT stage.

Existing guidelines (13–15) consider the purpose of NAs intervention during pregnancy to be to reduce the risk of mother-to-child transmission (MTCT) and to benefit the newborn. Thus, current guidelines all assume that the NAs can be stopped after delivery and lack guidance on postnatal treatment. However, a recent perspective (16) suggests that if these patients experience a significant reduction in HBsAg and/or HBeAg levels during pregnancy, they may benefit more from continued NAs therapy. The decision to continue AVT after delivery should be based on the individual circumstances of each patient.

At present, factors such as HBV genotyping, HBV DNA and HBeAg levels, ALT levels, and ongoing NAs therapy after delivery all influence HBeAg seroconversion during this time (9–11). Unfortunately, not all the influencing factors related to postnatal HBeAg seroconversion have been fully investigated and there currently exists no reliable combined predictor for determining the rate.

In this study, we analyzed prenatal and pregnant factors to develop a predictive model for estimating the rate of postpartum HBeAg seroconversion among HBeAg-positive women. This model can assist clinicians in identifying the population that would benefit most from targeted and sustained antiviral therapy, aiding in decision-making regarding optimal management for women.

## 2 Methods

### 2.1 Study design and patients

We retrospectively included HBeAg-positive HBV-infected mothers as the training cohort in the First Affiliated Hospital of Xi’an Jiaotong University (the largest tertiary university-affiliated hospital in the Northwest China, Xi’an, China) from January 2014 to December 2018 and prospectively enrolled patients as the validation cohort from January 2019 to December 2021.

Inclusion criteria encompassed postpartum women who met the following conditions: (1) aged between 18 and 45 years; (2) HBsAg-positive status for over 6 months; (3) HBeAg-positive status upon enrolment; (4) completion of blood tests at least at three distinct time points, including mid-pregnancy (occurring at 28 ± 4 weeks of gestation), delivery, and 2 years postpartum. Exclusion criteria were as follows: (1) coinfection with hepatitis C virus (HCV) or human immunodeficiency virus (HIV); (2) presence of comorbidities related to other liver diseases, including autoimmune liver disease, drug-induced liver injury, etc.; (3) decompensated cirrhosis; (4) any malignancies; (5) renal function abnormalities; (6) psychiatric disorders; (7) miscarriage occurring before 28 weeks; (8) intermittent use of NAs therapy during pregnancy or the postpartum period; (9) any other unsuitable conditions for study participation. A total of 489 cases were included in the training cohort and 94 cases were included in the validation cohort.

This study was approved by the Ethics Committee of the First Affiliated Hospital of Xi’an Jiaotong University (No. XJTU1AF2020LSYY-001-1) and adhered to the 1975 Declaration of Helsinki, relevant clinical practice guidelines (15, 17, 18), and local regulatory requirements. Due to the de-identification of information prior to inclusion in the analysis, the need for informed consent from study subjects was waived in the training set. The patients in the validation set all agreed to the study and signed the informed consent forms.

### 2.2 Treatment strategies

In alignment with established clinical guidelines, pregnant women who had not previously received NAs therapy before pregnancy were advised to initiate NAs therapy at approximately 28 ± 4 weeks of gestation if their HBV DNA level exceeded 5.3 log10 IU/mL, with the aim of preventing MTCT of HBV. For pregnant women with an HBV DNA level less than 5.3 log10 IU/mL, the decision regarding NAs therapy was made based on individual preference. Women who had undergone NAs therapy prior to pregnancy were transitioned to pregnancy class B medications [e.g., telbivudine (LDT), tenofovir disoproxil fumarate (TDF)] and were advised to continue this treatment following childbirth. If they agreed to continue NAs therapy after delivery, the women treated with TDF during pregnancy would continue TDF postpartum and the women treated with LDT during pregnancy would switch to entecavir (ETV) postpartum. If they did not agree to continue NAs therapy, they would be advised to discontinue antiviral drugs within 12 weeks after delivery.

### 2.3 Outcome

The primary endpoint in this study was the achievement of HBeAg seroconversion at 96 weeks postpartum, wherein HBeAg seroconversion was defined as the loss of HBeAg followed by the appearance of its corresponding antibody (HBeAb).

### 2.4 Potential predictive variables

We collected patients’ clinical data and laboratory findings. The clinical data included relevant demographic information such as age, family history, NAs therapy status, parity status, gestational week status, and pregnancy hepatitis flare [defined as ALT ≥2 times the upper limit of normal (ULN) during pregnancy, where ULN = 40 U/L]. Laboratory test results comprised liver function [alanine aminotransferase (ALT), aspartate transaminase (AST)] and HBV-related markers (HBsAg and HBV DNA levels). Laboratory tests were collected at least at mid-pregnancy and at delivery.

### 2.5 Variables selection and prediction model construction

We removed variables with missing values greater than 30% and ultimately included a total of 18 variables. These variables are: age, family history, NAs therapy before pregnancy, NAs therapy during pregnancy, NAs therapy after delivery, parity of ≥2 pregnancies, gestational week of ≥37, pregnancy hepatitis flare, ALT at mid-pregnancy and at delivery, AST at mid-pregnancy and at delivery, HBsAg level at mid-pregnancy and at delivery, HBV DNA level at mid-pregnancy and at delivery, HBsAg decline from mid-pregnancy to delivery and HBV DNA from mid-pregnancy to delivery.

The missingness in the dataset was as follows: HBV DNA level at mid-pregnancy (*n* = 10, 1.72%), HBsAg level at mid-pregnancy (*n* = 13, 2.23%), ALT at mid-pregnancy (*n* = 8, 1.37%), AST at mid-pregnancy (*n* = 10, 1.72%), HBV DNA level at delivery (*n* = 3, 0.51%), HBsAg level at delivery (*n* = 2, 0.34%), ALT at delivery (*n* = 6, 1.03%), AST at delivery (*n* = 6, 1.03%), gestational week of ≥37 (*n* = 7, 1.20%), parity of ≥2 pregnancies (*n* = 82, 14.07%) and family history ( *n* = 140, 24.01%). We then used multiple imputation for the remaining missing data with the R package “mice.” The mice package (19) employs the multiple imputation method, which predicts and fills in missing values by building predictive models based on variables with missing values and utilizing information from other variables. After multiple imputation, we compared the variables with missing values before and after imputation. Supplementary Table S2 shows that there is no significant difference among the variables (*p* > 0.05).

Candidate variables were choosed by the least absolute shrinkage and selection operator (LASSO) regression algorithm in R software in the training set. The selected variables were employed for multivariate regression analysis, with variables demonstrating *p*-values <0.05 from the multivariate regression analysis being integrated into the nomogram. The results included odds ratios (ORs) alongside their corresponding 95% confidence intervals (CIs).

### 2.6 Model performance assessment and validation

**Figure 1:**
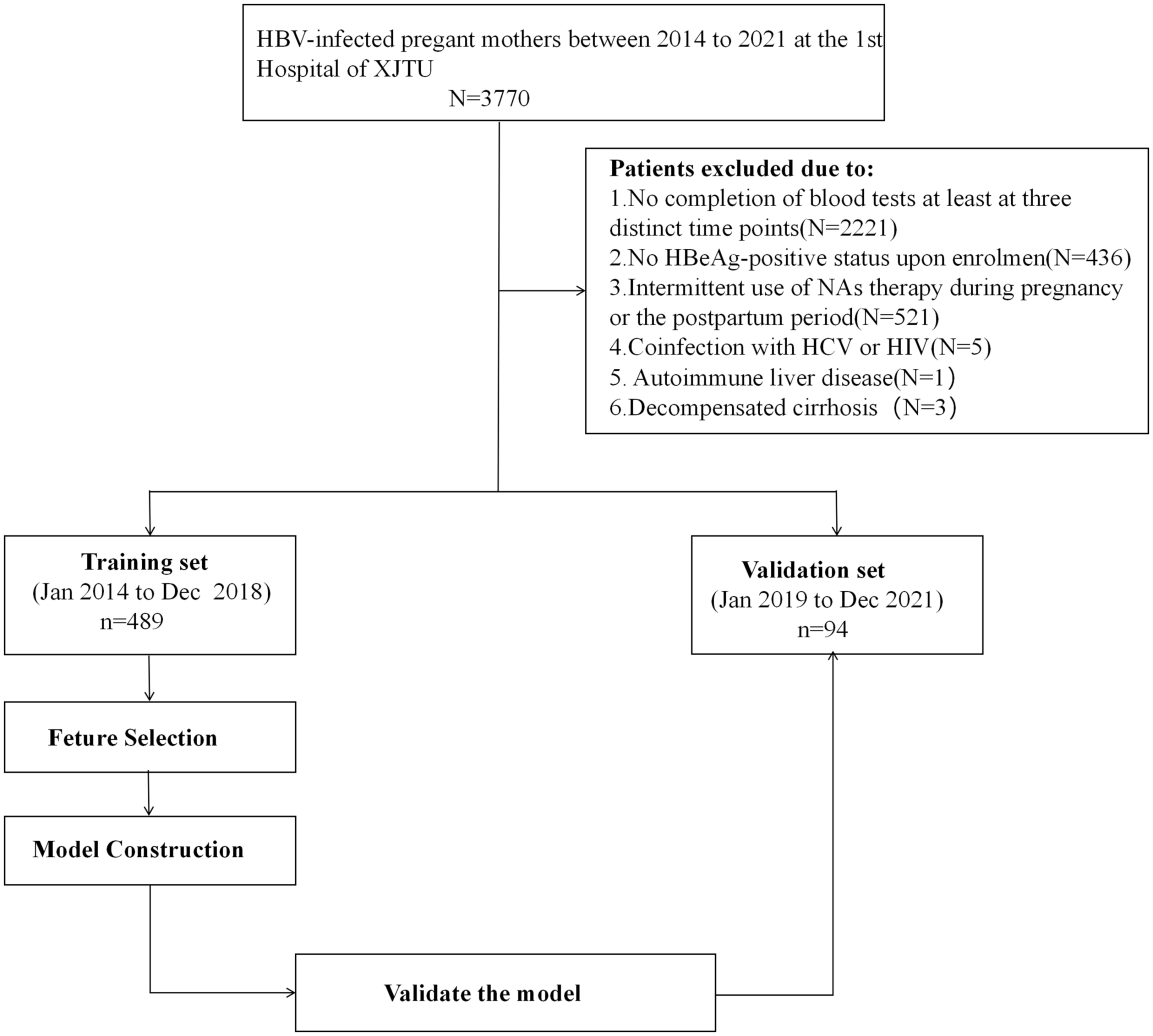
Flow diagram illustrating the model construction.

Model performance was evaluated in terms of discrimination, calibration and clinical utility. Discrimination ability was assessed using the area under the curve (AUC). Calibration was examined through the calibration plot along with the Hosmer–Lemeshow test. Clinical utility was determined using decision curve analysis (DCA) based on net benefits at different threshold probabilities. The model’s assessment was conducted in both the training set and the external validation set with regards to discrimination, calibration, and clinical value. Internal validations were done with 1,000 bootstrap resamples. The flow illustrating the model construction is shown in Figure 1.

### 2.7 Statistical analysis

Continuous variables conforming to a normal distribution were presented as means ± standard deviations (SDs) and compared using either student’s *t*-test or Levene’s test. Continuous variables deviating from a normal distribution were presented as medians [interquartile range (IQR)] and compared using the Mann–Whitney *U* test. Categorical data were expressed as *n* (%) and analyzed using either the chi-squared test or Fisher’s exact test. A significance level of *p* < 0.05 was deemed as statistically significant. All statistical analyses were performed using SPSS version 26.0 software (IBM, Armonk, NY, United States) and R software (version 3.6.3).

## 3 Results

### 3.1 Characteristics of patients enrolled in the study

This study included a total of 583 cases, with detailed information in Table 1. Among these patients, 21.78% (127/583) experienced HBeAg seroconversion after pregnancy. Significant differences were observed between the two groups in terms of NAs therapy before pregnancy, NAs therapy after delivery, pregnancy hepatitis flare, gestational week ≥37, all laboratory tests at mid-pregnancy, HBsAg and HBV DNA level at delivery (all *p* < 0.05).

**Table 1:** Characteristics of patients enrolled in the study.

Variables	Overall (*n* = 583)	HBeAg unseroconversion (*n* = 456)	HBeAg seroconversion (*n* = 127)	*p*-value
Clinical features				
Age, years	28 (26–31)	28 (26–31)	29 (27–31)	0.162
Family history, No. (%)	307 (52.66%)	248 (54.39%)	59 (46.46%)	0.113
NAs therapy before pregnancy, No. (%)	80 (13.72%)	50 (10.96%)	30 (23.62%)	<0.001
NAs therapy during pregnancy, No. (%)	559 (95.88%)	435 (95.39%)	124 (97.64%)	0.260
NAs therapy after delivery, No. (%)	316 (54.20%)	196 (42.98%)	120 (94.49%)	<0.001
Pregnancy hepatitis flare, No. (%)	79 (13.55%)	34 (7.46%)	45 (35.43%)	<0.001
Gestational week≥37, No. (%)	553 (94.85%)	437 (95.83%)	116 (91.34%)	0.043
Parity ≥2 pregnancies, No. (%)	221 (37.91%)	170 (37.28%)	51 (40.16%)	0.555
Laboratory tests at mid-pregnancy				
AST (U/L)	22.0 (18.0–29.0)	21.0 (17.9–26.0)	27.6 (21.0–46.9)	<0.001
ALT (U/L)	21.0 (15.0–31.0)	20.0 (15.0–28.1)	25.0 (19.0–49.4)	<0.001
HBsAg level (log10 IU/mL)	4.40 (3.68–4.67)	4.47 (3.95–4.72)	3.76 (3.30–4.37)	<0.001
HBVDNA level (log10 IU/mL)	7.70 (5.38–8.23)	7.88 (5.99–8.23)	6.77 (3.21–7.92)	<0.001
Laboratory tests at delivery				
AST (U/L)	22.0 (18.8–29.0)	22.0 (19.0–29.0)	22.0 (18.0–29.7)	0.874
ALT (U/L)	18.9 (14.0–28.0)	19.0 (14.0–27.0)	18.0 (13.3–29.0)	0.775
HBsAg level (log10 IU/mL)	4.23 (3.59–4.60)	4.38 (3.81–4.64)	3.56 (3.23–4.13)	<0.001
HBV DNA level (log10 IU/mL)	3.13 (2.00–4.15)	3.50 (2.38–4.39)	2.03 (1.32–2.90)	<0.001
Decline from mid-pregnancy to delivery				
HBsAg level (log10 IU/mL)	0.06 (−0.06–0.22)	0.06 (−0.05–0.20)	0.09 (−0.08–0.31)	0.233
HBV DNA level (log10 IU/mL)	3.82 (2.10–4.71)	3.84 (2.29–4.57)	3.73 (1.03–4.98)	0.727

NAs, nucleos(t)ide analogs; ALT, alanine aminotransferase; AST, aspartate aminotransferase; HBV, hepatitis B virus; HBsAg, hepatitis surface antigen. *p* < 0.05 was considered statistically significant.

Of the 583 individuals included, 489 were part of the training cohort and 94 were part of the validation cohort. There were no notable differences between the two groups in all variables except for ALT at mid-pregnancy and HBeAg seroconversion (*p* < 0.05). For detailed information (refer to Supplementary Table S1).

### 3.2 Screening for predictive factors

**Figure 2:**
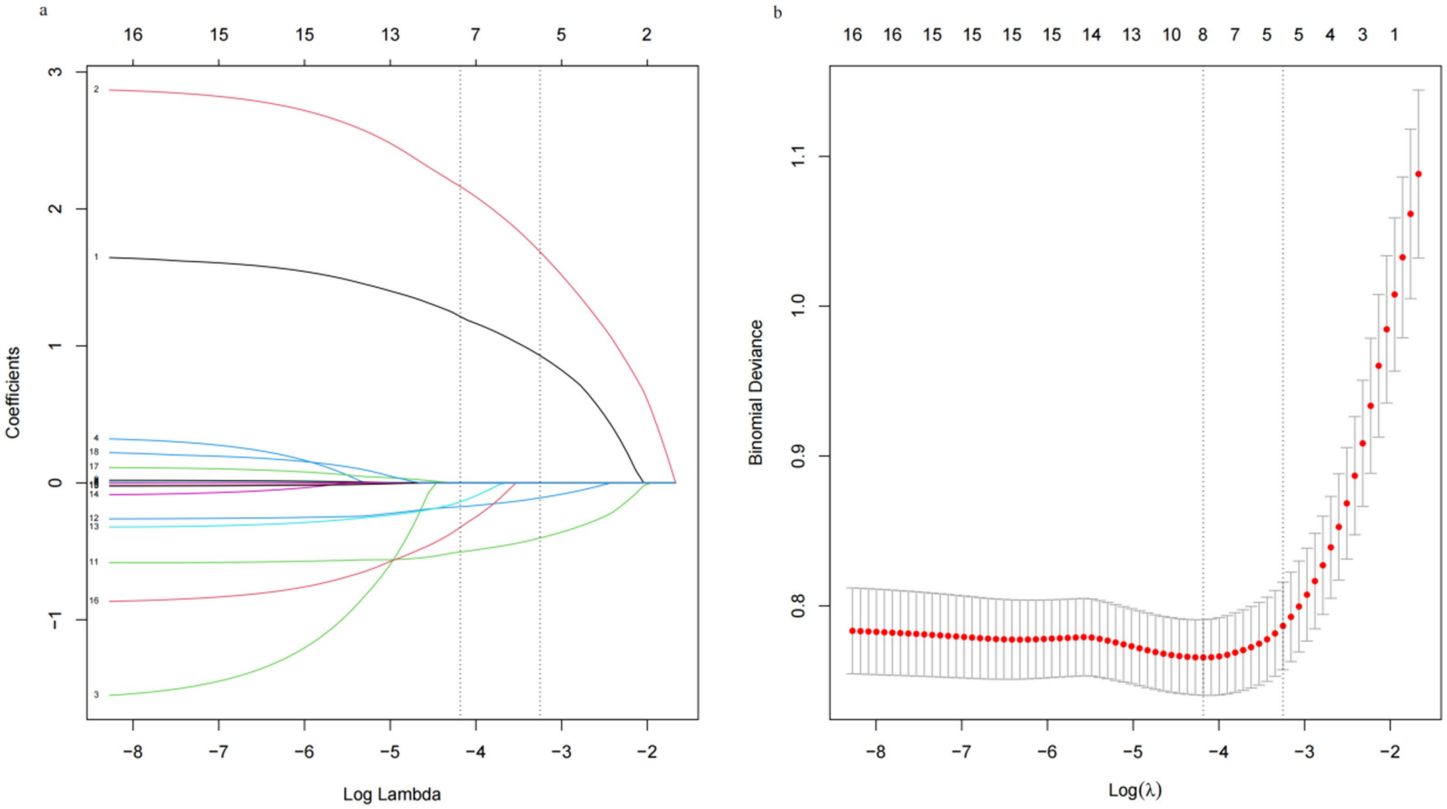
Least absolute shrinkage and selection operator (LASSO) regression analysis. (a) Log (lambda) value of the 18 features in the LASSO model. A coefficient profile plot was produced against the log (lambda) sequence. (b) In the LASSO model, the coefficient profiles of 18 texture features were drawn from the log (*λ*) sequence. Vertical dotted lines are drawn at the minimum mean square error (*λ* = 0.015) and the standard error of the minimum distance (*λ* = 0.039).

We implemented a LASSO regression algorithm for feature selection within the training set. The results revealed that, at a lambda value of standard error of the minimum distance (*λ* = 0.039), the initial 18 independent variables were reduced to 5 (Figure 2). To further mitigate the influence of confounding factors, we subjected these 5 independent variables to multivariate logistic regression analysis (refer to Supplementary Table S3). Ultimately, only pregnancy hepatitis flare, NAs therapy after delivery, HBsAg and HBV level at delivery were identified as significant characteristic factors (*p* < 0.05), as indicated in Table 2.

**Table 2:** Multivariate logistic regression analysis based on the selected four factors.

Predictors	*R*	SE	*p*-value	OR (95% CI)
HBsAg level (log10 IU/mL) at delivery	−0.599	0.203	0.003	0.549 (0.366–0.812)
HBV DNA level (log10 IU/mL) at delivery	−0.242	0.119	0.041	0.785 (0.619–0.986)
Pregnancy hepatitis flare	1.634	0.328	<0.001	5.122 (2.725–9.928)
NAs therapy after delivery	1.634	0.328	<0.001	15.051 (6.954–37.895)

*R*, regression coefficient; SE, standard error; OR, odds ratio; CI, confidence interval; NAs, nucleos(t)ide analogs; HBsAg, hepatitis B surface antigen; HBV, hepatitis B virus. *p* < 0.05 was considered statistically significant.

### 3.3 Model construction

**Figure 3:**
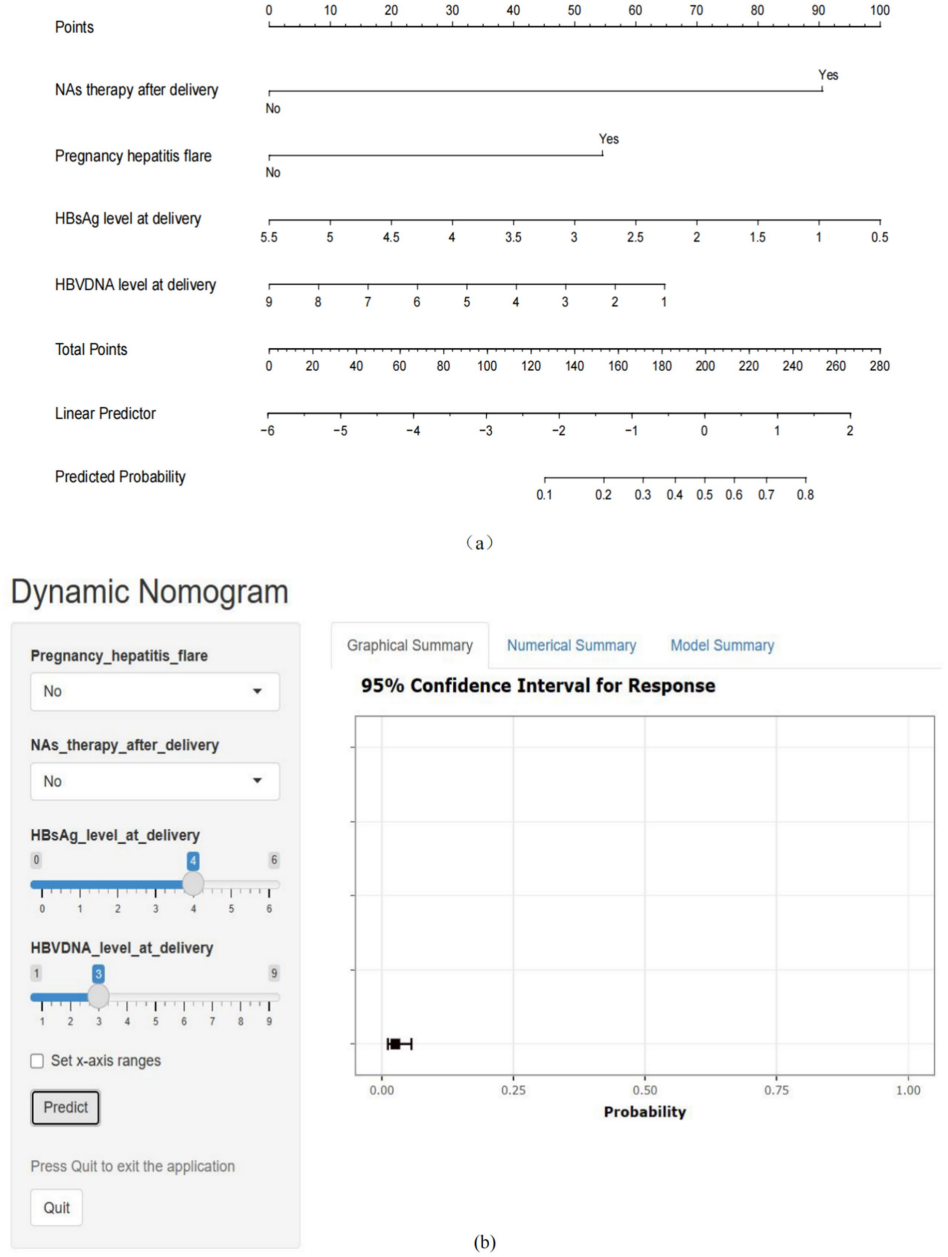
Nomogram for the prediction of HBeAg seroconversion after delivery. (a) Static nomogram for the prediction of HBeAg seroconversion after delivery. (b) Dynamic nomogram for the prediction of HBeAg seroconversion after delivery (https://wendyzhong.shinyapps.io/DynNomapp/).

The four factors derived from the multivariate logistic regression model were incorporated into the nomogram (Figure 3a). For each patient, a higher total score indicated a greater risk of HBeAg seroconversion. The dynamic nomogram is accessible at https://wendyzhong.shinyapps.io/DynNomapp/ (Figure 3b).

### 3.4 Model performance assessment

**Figure 4:**
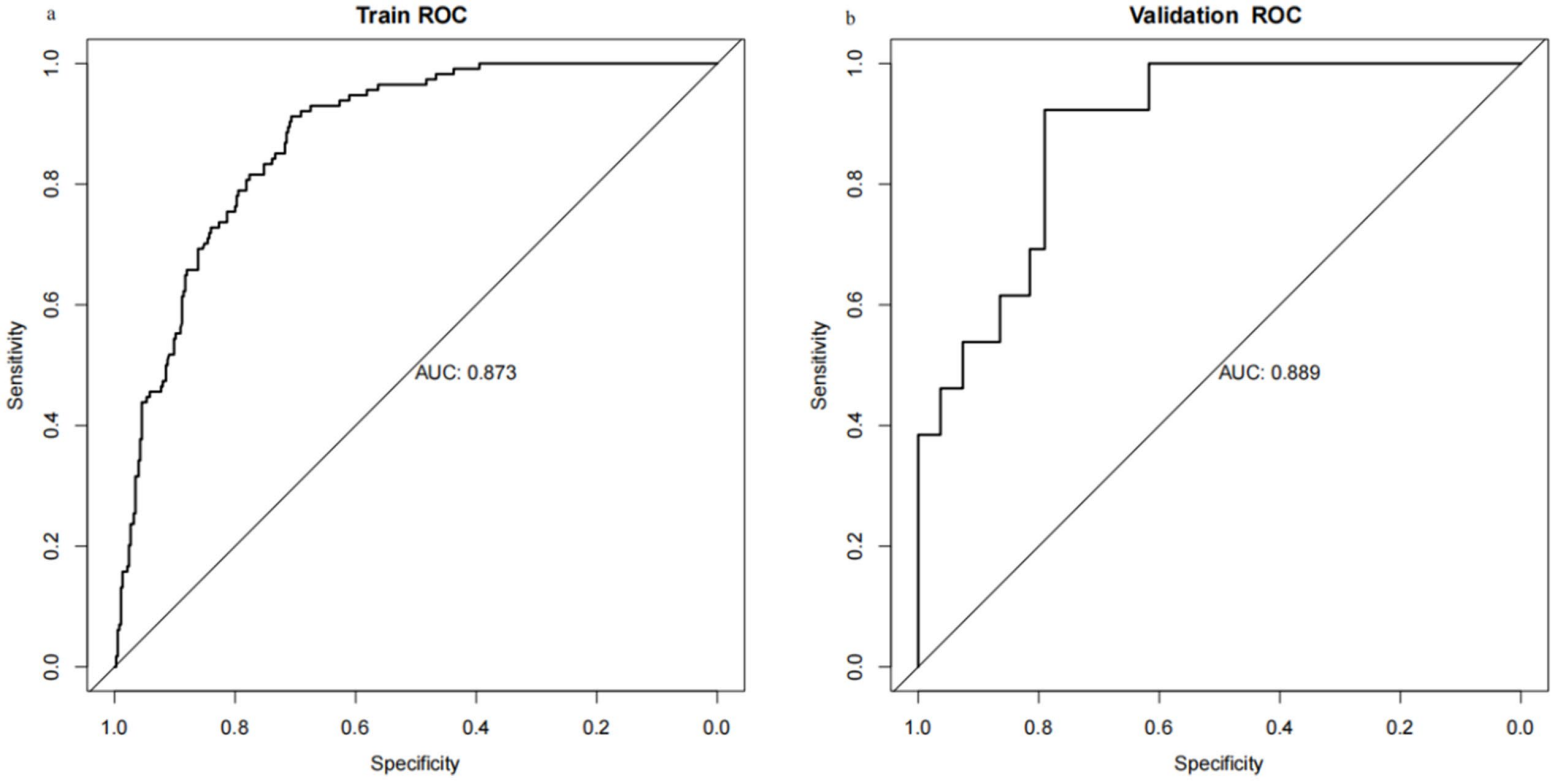
Receiver operating characteristic (ROC) curves for training set and validation set. (a) Training cohort. (b) Validation cohort.

**Figure 5:**
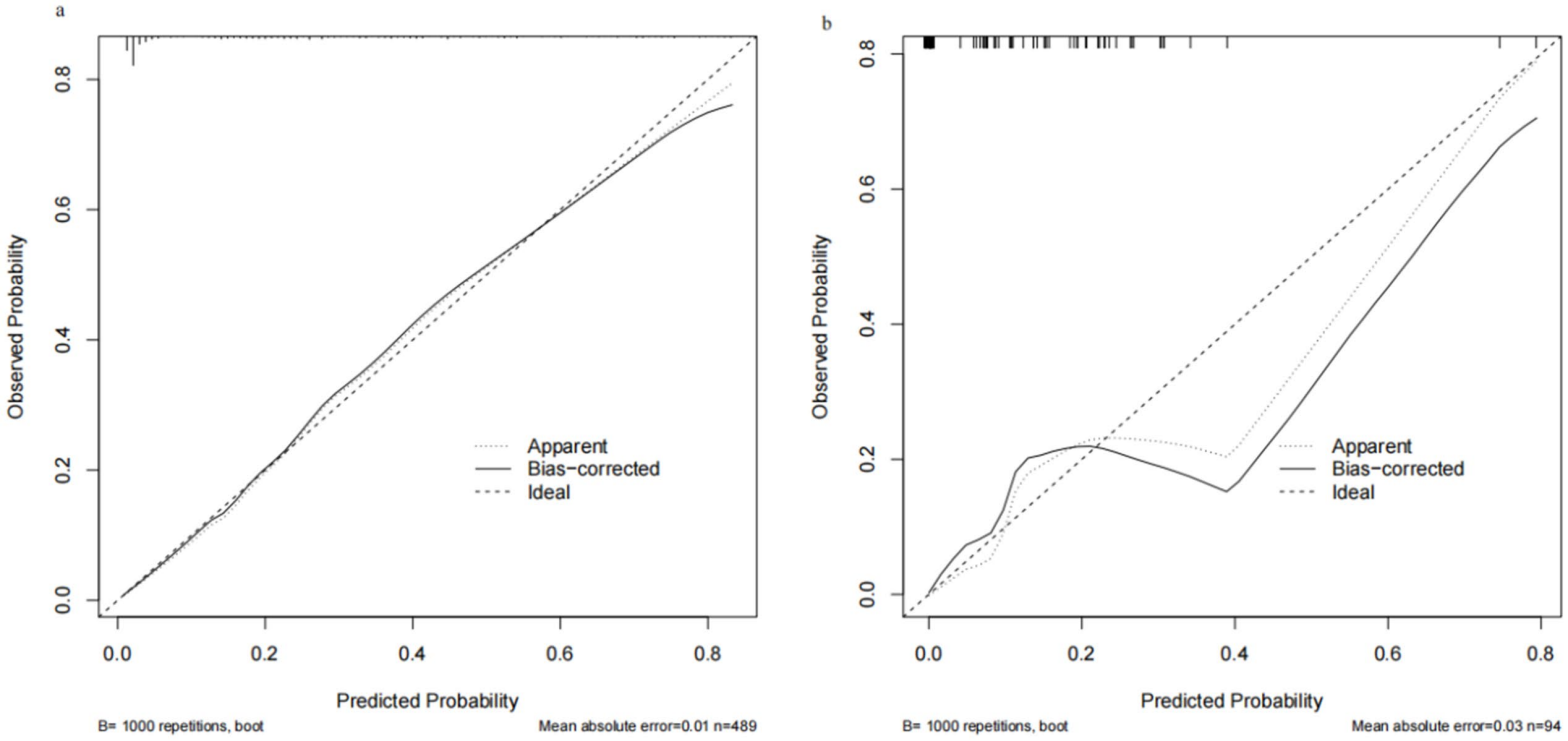
Calibration curve for training set and validation set. (a) Training cohort. (b) Validation cohort.

**Figure 6:**
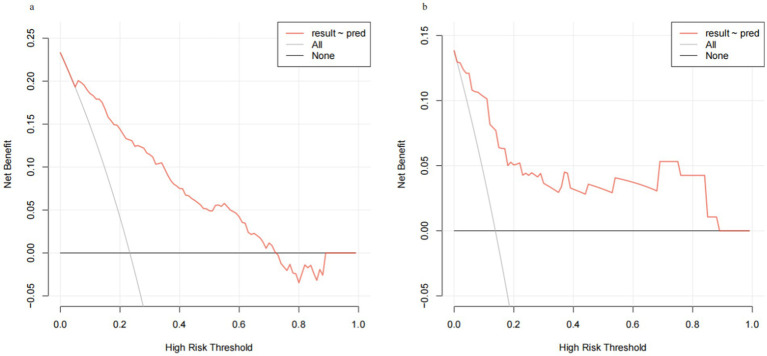
Decision curve analysis (DCA) for training set and validation set. (a) Training cohort. (b) Validation cohort.

Through internal bootstrap validation with 1,000 resamples, the mean AUC of the nomogram was determined to be 0.873 (95% CI: 0.839–0.904) (Figure 4a), underscoring its commendable discriminatory accuracy. The Hosmer–Lemeshow test yielded a *p*-value of 0.400. The calibration curve closely approximated the ideal diagonal line (Figure 5a). Furthermore, DCA revealed a significantly improved net benefit associated with the predictive model (Figure 6a). In the validation set, the AUC was 0.889 (95% CI: 0.801–0.953) (Figure 4b). Additionally, the model exhibited robust consistency, and the calibration curve for the validation set was in close proximity to the ideal diagonal line (Figure 5b). The p-value of the Hosmer–Lemeshow test in the validation set was 0.612. Furthermore, DCA demonstrated a significant net benefit associated with the predictive model, consistent with the findings in the training set (Figure 6b). Collectively, these data underscore the substantial potential of our nomogram for informing clinical decision-making.

## 4 Discussion

To our knowledge, this is the first group aiming at pregnant women with HBV and tracking them for up to 2 years after childbirth in northwest China. In this retrospective cohort study, about 21.78% patients experienced HBeAg seroconversion within 96 weeks after pregnancy. The rate of HBeAg seroconversion among mostly IT women in this group was higher than the rates seen in non-pregnant, IT groups as reported previously (18, 20).

This phenomenon may be related to postnatal immune reconstitution. During pregnancy, the immune system experiences suppression. Following delivery, there is a significant hormonal shift, leading to the cessation of immunosuppression (21). This results in an increase in CD8^+^ T cell numbers, the recovery of HBV-specific T-cell responses previously affected by pregnancy-related dysfunction, and a notable elevation in various cytokines (22). Consequently, some women who were either in or near the IT phase before pregnancy will transfer transition to the immune-clearing phase postpartum. Multiple observational clinical studies (11, 12, 23) have provided evidence that maintaining AVT postpartum in this population yields higher rates of HBeAg seroconversion in comparison to the general IT population. Consequently, the postpartum period emerges as an opportune window for HBV treatment and disease regression. The development of the current model offers a practical tool for precise and efficient identification of this specific population.

It encompasses four primary variables: pregnancy hepatitis flare, NAs therapy after delivery, HBsAg and HBV DNA level at delivery. The predictive model has demonstrated robust performance, boasting exceptional discrimination, calibration, and certain clinical advantages. Notably, the high AUC values obtained in the external validation set suggest that the model can be widely and accurately applied with large sample sizes.

Lower prenatal levels of HBsAg and HBV DNA predicted higher rates of postnatal HBeAg seroconversion. This association arises from the fact that the HBV DNA and HBsAg level reflect both the quantity and transcriptional activity of cccDNA within hepatocytes, as well as the immune system’s control over HBV (24). HBsAg and HBV DNA levels in non-pregnant cohorts have been widely used for prediction of HBeAg seroconversion after AVT (25, 26). And in the pregnant population, several studies have shown similar results to ours (11, 27, 28).

In addition, HBeAg titer and its decline have frequently been utilized to forecast HBeAg seroconversion (12, 26). However, it is important to note that, unlike HBsAg and HBV DNA, which have internationally recognized quantitative standardized units for detection, there is currently no universally accepted quantitative unit of measurement for HBeAg. The lack of a standardized measure complicates the comparison and interpretation of results across different units (29). Additionally, HBeAg titer is not routinely tested for HBV-infected patients in many hospitals, so we did not include this variable in our study.

At present, almost all existing guidelines (13, 14) suggest initiating AVT for non-pregnant patients with HBV viral load ≥2,000 IU/mL and ALT levels ≥1ULN. Because pregnancy itself can also affect liver function and lead to slightly elevated ALT levels even in pregnant women without HBV (30), whether this threshold for AVT should be applied in the pregnant population is debatable. The Chinese guideline (15) recommends close monitoring when ALT >2ULN. If ALT levels continue to rise after ruling out other potential causes, and when combined with other indicators, it suggests active hepatitis. AVT with TDF is advised for individuals meeting the criteria for CHB treatment, with continued therapy post-delivery. In this study, to account for the impact of pregnancy on liver function, we defined flare as ALT >2ULN. We observed that individuals experiencing a flare during pregnancy were more likely to achieve negative results after delivery, consistent with findings from previous studies (11, 12, 23).

It may not come as a surprise that women with high ALT levels are more prone to HBeAg seroconversion (31). The new finding is that when flare does not occur or is not monitored during pregnancy, the combination of the other 3 factors can still be predictive of the rate of HBeAg conversion in the postpartum period, thus providing optimal therapeutic management in the postpartum period.

The final predictor is administration of NAs therapy after delivery. In contrast to the three previously factors, the decision to continue NAs therapy after childbirth is the only factor that can be actively managed by either the patient or the physician. At present, typically IT patients have not been recommended to receive AVT due to the perception that they were not susceptible to progressive liver disease during this phase of CHB infection.

As most pregnant women are at or near the IT stage, almost all current guidelines suggest discontinuing AVT postpartum for this group. However, there has been recent debate regarding this practice. It’s been suggested that pregnant women whose viral markers have significantly decreased during pregnancy may benefit from AVT after delivery, even if their liver function remains normal throughout gestation, due to the restoration of immunity in the postnatal period.

Thus, when deciding whether a patient should stop NAs therapy after childbirth, we can consider the likelihood of achieving HBeAg seroconversion after delivery. We first collect data on pregnancy hepatitis flare, NAs therapy after delivery, HBsAg and HBV DNA level at delivery. Using this data, the predictive model calculates the probability of HBeAg seroconversion with or without continuing NAs therapy after delivery, which helps identify whether the patient is a suitable candidate for ongoing AVT after giving birth, enabling the provision of precise and effective recommendations for postnatal HBV-infected mothers’ treatment regimens.

This study has several limitations. Firstly, the training cohort is retrospective, which means that loss to follow-up and incomplete data sets can affect interpretation. Secondly, it is challenging to accurately find the exact time of HBeAg seroconversion. Therefore, in line with previous studies, we chose to use 2 years postpartum as the study endpoint (15, 18, 21). Thirdly, due to the lack of consensus on postpartum follow-up procedures, the blood tests are frequently non-regimented. We did not include postpartum laboratory test results in our analyses. Additionally, the included risk factors primarily focus on the middle and late stages of pregnancy, neglecting the use of pre-pregnancy and early pregnancy data. Furthermore, the study included a small sample exclusively from the Chinese Han population and was validated in the same hospital. Future research should include multi-center validation and large-scale prospective studies from pre-pregnancy to postpartum to enhance the robustness of the findings.

## 5 Conclusion

We have developed a predictive model for postpartum HBeAg seroconversion in women with chronic HBV infection who are HBeAg-positive, based on the identified predictors. This model relies on identified predictors to effectively pinpoint the subset of women likely to undergo this change after receiving antiviral therapy post-delivery. This helps in making informed decisions regarding the most suitable management for these women.

## Data Availability

The raw data supporting the conclusions of this article will be made available by the authors, without undue reservation.
