# Supplementary material for: Development and validation of a multivariable nomogram predictive of hepatitis B e antigen seroconversion after pregnancy in hepatitis B virus-infected mothers
Source: Front Med (Lausanne). 2024 Oct 21;11:1428569. doi: 10.3389/fmed.2024.1428569 (PMC11532139; doi:10.3389/fmed.2024.1428569)
Supplement: Supplementary file 1 [file Table_1.DOCX]

**Supplemental Online Content**

**eTable1 Characteristics of patients in training cohort and validation cohort**

**eTable2 Difference characteristics between the pre- and post-multiple imputation data**

**eTable3** **Multivariate logistic regression analysis based on the selected five factors**

**eTable 1 Characteristics of patients in** **training cohort and** **validation cohort**

| **Variables** | **Overall**  **(n=583)** | **Training cohort**  **(n=489)** | **Validation cohort**  **(n=94)** | **P-value** |
| --- | --- | --- | --- | --- |
| **Clinical features** |  |  |  |  |
| Age, years | 28(26–31) | 28(26–31) | 29(26–31) | 0.771 |
| Family history, No.(%) | 307(52.66%) | 262(53.58%) | 45(47.87%) | 0.310 |
| NAs therapy before pregnancy, No.(%) | 80(13.72%) | 67(13.70%) | 13(13.83%) | 0.974 |
| NAs therapy during pregnancy, No.(%) | 559(95.88%) | 472(96.52%) | 87(92.55%) | 0.088 |
| NAs therapy after delivery, No.(%) | 316(54.20%) | 262(53.58%) | 54(57.45%) | 0.491 |
| Pregnancy hepatitis flare, No.(%) | 79(13.55%) | 71(14.52%) | 8(8.51%) | 0.119 |
| Gestational week≥37, No.(%) | 553(94.85%) | 462(94.48%) | 91(96.81%) | 0.451 |
| Parity ≥2 pregnancies, No.(%) | 221(37.91%) | 189(38.65%) | 32(34.04%) | 0.399 |
| **Laboratory tests at mid-pregnancy** |  |  |  |  |
| AST(U/L） | 22.0(18.0–29.0) | 22.0(18.0–29.0) | 20.0(17.8–26.8) | 0.269 |
| ALT(U/L） | 21.0(15.0–31.0) | 21.0(16.0–32.0) | 19.0(14.0–26.0) | 0.017 |
| HBsAg level(log10 IU/mL) | 4.40(3.68–4.67) | 4.40(3.65–4.70) | 4.40(3.78–4.62) | 0.743 |
| HBVDNA load (log10 IU/mL) | 7.70(5.38 – 8.23) | 7.70(5.45–8.23) | 7.67(4.86–8.23) | 0.718 |
| **Laboratory tests at delivery** |  |  |  |  |
| AST(U/L） | 22.0(18.8–29.0) | 22.4(19.0–30.0) | 21.0(18.0–27.0) | 0.048 |
| ALT(U/L） | 18.9(14.0–28.0) | 19.0(14.0–28.0) | 17.0(12.3–26.8) | 0.107 |
| HBsAg level(log10 IU/mL) | 4.23(3.59–4.60) | 4.21(3.57–4.60) | 4.28(3.62–4.59) | 0.750 |
| HBV DNA load (log10 IU/mL) | 3.13(2.00–4.15) | 3.21(2.00–4.23) | 2.78(2.00–4.01) | 0.188 |
| **Decline from mid-pregnancy to delivery** |  |  |  |  |
| HBsAg level(log10 IU | 0.06(-0.06–0.22) | 0.06(-0.05–0.230) | 0.07(-0.12–0.20) | 0.341 |
| HBV DNA load (log10 IU/mL) | 3.82(2.10–4.71) | 3.82(2.01–4.69) | 3.83(2.38–4.77) | 0.623 |
| **HBeAg seroconversion** | 127(21.78%) | 114(23.31%) | 13(14.89%) | 0.041 |

**Abbreviations:**NAs,nucleos(t)ide analogs;ALT,alanine aminotransferase;AST,aspartate aminotransferase; HBV, hepatitis B virus;HBsAg,hepatitis surface antigen;HBeAg,hepatitis B e antigen.

P＜0.05 was considered statistically significant.

**eTable 2. Difference characteristics between the pre- and post-multiple imputation data**

| **Variables** | **Before**  **imputation** | **After**  **imputation** | **P** |
| --- | --- | --- | --- |
| HBV DNA level at mid-pregnancy | 7.70(5.37–8.23) | 7.70(5.38–8.23) | 0.912 |
| HBsAg level at mid-pregnancy | 4.41(3.71–4.67) | 4.40(3.68–4.67) | 0.826 |
| ALT at mid-pregnancy | 21.0(15.0–31.0) | 21.0(15.0–31.0) | 0.922 |
| AST at mid-pregnancy | 22.0(18.0–29.0) | 22.0(18.0–29.0) | 0.944 |
| HBV DNA level at delivery | 3.13(2.00–4.15) | 3.13(2.00–4.15) | 0.971 |
| HBsAg level at delivery | 4.23(3.60–4.60) | 4.23(3.59–4.60) | 0.953 |
| ALT at delivery | 19.0(14.0–28.0) | 18.9(14.0–28.0) | 0.908 |
| AST at delivery | 22.0(18.8–29.0) | 22.0(18.8–29.0) | 0.974 |
| Gestational week of ≥37 | 546(94.79%) | 553（94.85%） | 0.962 |
| Parity of ≥2 pregnancies | 189（37.72%） | 221（37.91%） | 0.951 |
| Family history | 236（53.27%） | 307（52.66%） | 0.845 |

**Abbreviations:**ALT,alanine aminotransferase;AST,aspartate aminotransferase; HBV, hepatitis B virus;HBsAg,hepatitis surface antigen.

P＜0.05 was considered statistically significant.

**eTable 3 Multivariate logistic regression analysis based on the selected five factors**

| **Predictors** | **R** | **SE** | **P-value** | **OR(95%CI)** |
| --- | --- | --- | --- | --- |
| AST(U/L) at mid-pregnancy | 0.005 | 0.004 | 0.256 | 1.005(0.998-1.015) |
| HBsAg level(log10 IU/mL) at delivery | -0.589 | 0.202 | 0.004 | 0.555(0.370-0.820) |
| HBVDNA level(log10 IU/mL) at delivery | -0.234 | 0.118 | 0.049 | 0.792(0.624-0.995) |
| Pregnancy hepatitis flare | 1.371 | 0.385 | <0.001 | 3.940(1.855-8.466) |
| NAs therapy after delivery | 2.682 | 0.425 | <0.001 | 14.610(6.769-36.689) |

**Abbreviations:**R,regression coefficient;SE,Standard error;OR,odds ratio;CI,confidence interval;NAs,nucleos(t)ide analogs;HBsAg,hepatitis B surface antigen;AST,aspartate aminotransferase; HBV, hepatitis B virus.

P＜0.05 was considered statistically significant.
